# Dietary Omega-6/Omega-3 Polyunsaturated Fatty Acid (PUFA) and Omega-3 Are Associated With General and Abdominal Obesity in Adults: UK National Diet and Nutritional Survey

**DOI:** 10.7759/cureus.30209

**Published:** 2022-10-12

**Authors:** Salwa A Albar

**Affiliations:** 1 Food and Nutrition Department, King Abdulaziz University, Jeddah, SAU

**Keywords:** waist-to-height ratio, relative fat mass, waist circumference, omega-6/omega-3 ratio, abdominal obesity, obesity, omega-3, omega-6, dietary, polyunsaturated fatty acids

## Abstract

Introduction

The link between dietary fats and obesity is still controversial, as in Western diets the percentage of energy from total fat has decreased while the intake of omega-6 has increased, and omega-3 decreased. These changes have corresponded with a significant increase in the prevalence of obesity.

Objective

This study aims to examine the association of percentages of energy intake (EI) from omega-3 and omega-6 and Σω-6/Σω-3 ratio with BMI and two proxy indicators of central obesity (waist circumference [WC], waist-to-height ratio [WHtR]) and relative fat mass (RFM) estimator of whole-body fat.

Design

A representative sample of 3,733 adults was used from the UK National Diet and Nutrition Survey Rolling Programme (2008/09-2018/19). An estimated four-day food record was used to calculate dietary intake. Regression models were used to verify the association of omega-3 and omega-6 and quintiles of Σω-6/ Σω-3 ratio with general and abdominal obesity with adjustment for important confounders. A p-value of <0.05 represented statistical significance.

Results

The findings of this study show that the average ratio of Σω-6/Σω-3 was 5.5:1 ± 2. There was a significant association between the ratio of Σω-6/Σω-3 and BMI, WC, WHtR and RFM. However, the percentage of total EI from total fat was only significant with BMI, while the percentage of EI from omega-3 was negatively associated with WC, WHtR and RFM. No association was found between the percentage of EI from omega-6 and general or abdominal obesity.

Conclusion

The effect of Σω-6/Σω-3 may be largely driven by a deficiency in the intake of omega-3. Omega-6 and omega-3 should be listed as such in national surveys instead of polyunsaturated fatty acid (PUFA). Meeting recommended levels of omega-3 and lowering Σω-6/Σω-3 are imperative to establish healthier dietary patterns and prevent obesity.

## Introduction

The epidemic of overweight and obesity is a significant clinical and public health burden, both internationally and in the UK. By 2030, if recent trends continue to persist, the absolute numbers are projected to reach 2.16 billion overweight and 1.12 billion obese adults [1]. In 2019, 28% of adults in England were obese and 36% were overweight, making around two-thirds (a total of 64.2%) either overweight or obese [2].

Obesity is an excessive accumulation of adipose tissue. It is associated with a higher risk of metabolic diseases and other comorbidities [3]. According to epidemiological studies, obesity increases the risk of other health conditions, including metabolic syndrome [4], cardiovascular diseases, type 2 diabetes, kidney disease, different types of cancer, and musculoskeletal complications [2]. Although genetic factors play a role in the development of obesity [5,6], dietary fat intake [7] and its types [5] have been suggested to play an essential role in obesity [8]. Dietary fat may interact with genotype to increase the risk of obesity and metabolic syndrome (MetS) [5]. However, the effects of dietary fatty acids on health are influenced greatly by the kind and proportion of the fatty acids [9].

Thus far, the relationship between dietary fats and obesity is still controversial [8,10]. Research has shown that saturated fatty acids (SFAs) have positive [9] and negative [7] associations with weight gain. Similar relationships have been found with the intake of monounsaturated fatty acids (MUFAs) where positive [6] and negative associations [11] in developing obesity have been found. No significant association was found between the amount or type of dietary fat (SFAs, MUFAs, and polyunsaturated [PUFAs]) and subsequent weight change in a large prospective study using country-specific food-frequency questionnaires [10]. This discrepancy result may suggest that the total fatty acid is not sold enough to reveal a consistent relationship between dietary fat and developing obesity.

In the past three decades, the proportion of total intake of fat and SFAs has continuously decreased in Western diets, while the intake of omega-6 PUFA has increased [12] and the intake of omega-3 PUFA has decreased [12]. These changes in the composition of fatty acids ran in parallel with a significant increase in the prevalence of overweight and obesity [7,13]. Different experimental studies have shown that omega-6 and omega-3 have different effects on body fat through different mechanisms of adipogenesis [14], lipid homeostasis, inflammation [15] and brain-gut adipose tissue axis [16]. Omega-3 PUFA is considered a potential protective nutrient against the cardiometabolic risks associated with obesity [17]. Omega-3 PUFA can alter cell membrane function and metabolism, decreasing circulating inflammatory markers [18]. Some studies have found that a low omega-3 index has been linked to a high level of body mass index [19,20]. A higher intake of omega-3 PUFA improves managing obesity, which is considered a chronic state of inflammation [18]. In contrast, intake of omega-6 PUFA increases inflammation in metabolic tissues [21], with higher intakes linked to an increase in the incidence of obesity [13]. High concentrations of omega-6 in red blood cell membranes have been associated with a high risk of obesity [22].

Data from food commodities have shown an increasing imbalance in the ratio of omega-6/omega-3 in the food supply over time [23]. Over the last 100 years, an enormous production of vegetable oils that contain a high amount of omega-6 has taken place [24]. Changing animal feeds in modern agriculture has decreased the omega-3 fatty acid content of animal meat, eggs and even fish [13]. This has resulted in very high amounts of omega-6 in the food supply for the first time in human history [25]. Some studies have indicated that ratios of omega-6/omega-3 in food commodities have reached above 20:1 [26]. Moreover, in Western countries, it has been reported that the intake of omega-3 is insufficient and often accompanied by a high dietary Σω-6/Σω-3 PUFA ratio. Findings from the National Health and Nutrition Examination Survey (NHANES) that considered 24 dietary intakes have shown ratios of omega-6 to omega-3 dietary intake to be closer to 10:1 [27].

Although omega-6 and omega-3 are both essential for human health and must be obtained from a person’s diet, absolute intake and the Σω-6/Σω-3 ratio required to attain optimal health benefits are figures considered somewhat controversial. As a result, there has been a failure to consider such intake in the context of total daily fat as a percent of total energy intake (EI) [28]. Very few observational and clinical studies have examined the association between the composition of PUFAs (particularly omega-6, omega-3, and Σω-6/Σω-3 ratio) and the specific parameter of obesity using a small sample size [29]. Therefore, this study aims to investigate the association of the percentages of EI obtained from omega-3 and omega-6 and Σω-6/Σω-3 ratio with BMI, and two proxy indicators of central obesity (waist circumference [WC], the waist-to-height ratio [WHtR]), as well as the relative fat mass (RFM) estimator of whole-body fat.

## Materials and methods

National diet and nutritional survey rolling program (NDNS-RP)

NDNS-RP data were obtained from the UK Data Service at the University of Essex. The NDNS is a cross-sectional survey, designed to assess the nutritional status of the general population in the UK [30]. This study used combined data from years 1 to 11 (2008/09-2018/19) as high-quality, nationally representative data. Methods used in the NDNS-RP are kept consistent over time. More detailed information regarding the study’s design, sampling methodology, and dietary methods used is given in published reports [30,31]. For years 1 to 5 the ethical approval for the NDNS-RP was obtained from the Oxfordshire Research Ethics Committee (Ref. No. 07/H0604/113), then it was obtained from the Cambridge South NRES Committee (Ref. No. 13/EE/0016) [31]. Pregnant women, those with information missing related to height or weight, and WC were excluded. Also, participants with EI less than 500 or over 3,500 kcal (to avoid over or under dietary intake reporting [32]) were excluded from the analyses. A total of 3,733 adults who participated in the NDNS-RP from 2008 to 2019 have been included in this study.

Dietary method

An estimated four-day food record was used in the NDNS-RP. Participants were asked to keep a record of everything they ate and drank over four consecutive days, both inside and outside the home. Trained interviewers visited each participant three times to review the diary in order to guarantee the accuracy of the data. All interviewers followed standard protocols and explained how to report food intake and how to describe food and drink consumption in more detail. All food diaries were reviewed and coded by trained dietitians.

Anthropometric measurement and abdominal obesity

Height and weight were measured in the first interview visit. A portable stadiometer was used to measure height to the nearest 0.1 cm and weight scales were used to measure to the nearest 0.1 kg. Body mass index (BMI) was calculated (weight [kg]/height [m^2^]) and the BMI value was interpreted based on World Health Organization (WHO) and National Institute for Health and Clinical Excellence (NICE) classifications, where BMI < 18.5 kg/m^2^ is underweight, 18.5≤BMI≤24.9 kg/m^2^ is normal, 25≤BMI≤29.9 kg/m^2^ is overweight, and BMI≥30 kg/m^2^ is obese [33]. To measure waist circumference (WC) a tape in mm was used, and all measurements were taken to the nearest millimeter. Also, the WHtR was calculated by dividing WC (cm) by height (cm). WHtR is a simple screening risk assessment tool that helps to identify people at early health risk associated with central obesity, such as cardiovascular disease (CVD) risk factors [34,35]. To estimate whole-body fat percentage among adults, RFM was calculated as follows: 64 − (20 × height/WC) + (12 × sex); sex = 0 for males and 1 for females, RFM reduced total obesity misclassification overall [36].

Statistical analysis

Descriptive and summary statistics, including mean, ± standard deviations (SD); N, % were used to describe the general characteristics of the sample and those after being stratified by gender. A Pearson Chi-Square test was used to investigate the differences between males and females in the categorical general characteristics. A two-sample t-test (a two-tailed test) was used for continuous characteristics. Also, summary statistics for the mean, ± SD of EI and macronutrients, Σω-6/ Σω-3 PUFAs ratio, and their contribution to total EI were calculated and presented. One-way analysis of a variance regression model-1 was used to assess differences in the proportion of EI from macronutrients and Σω-6/ Σω-3 PUFA between genders, adjusted for confounders (total EI, BMI, age, household income, ethnicity, and smoking). To identify an association between the quintiles of Σω-6/ Σω-3 PUFA ratio as an independent variable and BMI, WC, WHtR, and RFM as dependent variables, one-way analysis of a variance regression model-2 was used, after adjusting for confounders (age, gender, EI, ethnicity, total household income, and smoking). A statistical significance level of P < 0.05 was used and the analyses were carried out using Stata statistical software release 15 (Stata Corporation) [37].

## Results

Sample characteristics

A total of 3,733 participants were included in this study. A total of 58.3% of them were female and their average age was 43.1±12.5 for the females and 44.5 ±12.7 for the males. The differences in age were significant between genders. The majority of participants were from European ethnic groups (92.5%). There were significant differences in the proportion of household income between males and females. Furthermore, there were significant differences between the proportion of males and females in the BMI category (p < 0.001), as well as in the mean of abdominal obesity variables between males and females (Table 1). A total of 98.9% of the sample completed the four-day food record and 97% of participants did not follow any specific diet. In total, 24.3% of the sample were current smokers and there were significant differences in the proportion of smoking status between males and females (p < 0.05).

**Table 1:** General characteristics of adults (19-65 years) who participated in the study ^†^Significant differences between the proportion of females and males in the categorical data were assessed by using the Chi-2 test. ^‡^Mean differences between females and males in continuous demographic and anthropometric variables were assessed by using regression model. *There was 4.9% of participants refused to answer/no answer.

Demographic and anthropometric variables	All Participants (n= 3,733)		Females (n=2,176)		Males (n=1,557)		P-value
	Mean	±SD	Mean	±SD	Mean	±SD	
Age (years)^‡^	43.7	12.6	43.1	12.5	44.5	12.7	0.001
Ethnicity (n, %)^ †^							
European	3,453	92.5	2,009	92.3	1,445	92.8	0.585
Asian and other groups	279	7.5	167	7.7	112	7.2	
Anthropometric measures^‡^							
Height (cm)	168.1	9.2	162.6	6.3	175.8	6.9	< 0.001
Weight (kg)	77.8	16.8	72.3	15.6	85.6	15.3	< 0.001
BMI (kg/m^2^) (n, %)^ †^							
Underweight	41	1.1	29	1.3	12	0.8	< 0.001
Normal weight	1,316	35.3	868	39.8	448	28.8	
Overweight	1,339	35.8	667	30.7	672	43.2	
Obese	1,037	27.8	612	28.1	425	27.2	
Abdominal obesity^‡^							
Waist circumference (cm)	92.2	14.3	88.3	14.0	97.7	12.8	< 0.001
Relative fat mass (RFM)	33.7	7.7	38.3	5.9	27.4	4.8	< 0.001
Waist to height ratio WHtR	0.55	0.1	0.54	0.1	0.56	0.1	< 0.001
Total House income (n, %)^ †*^							
Under £14,999	737	20.7	482	23.3	255	17.2	< 0.001
£15,000 - £29,999	1,051	29.7	642	31.1	409	27.6	
£30,000 – £44,999	768	21.6	421	20.4	347	23.4	
£45000 – £99,999	856	24.1	436	21.1	420	28.4	
£100,000 or more	135	3.8	85	4.1	50	3.4	
Four food diary (Yes, n, %)^†^	3,693	98.9	2,147	98.7	1,546	99.3	0.067
Following special Diet (Yes, n, %)^ †^							
Vegetarian	107	2.9	79	3.6	28	1.8	0.001
vegan	8	0.2	7	0.3	1	0.1	
neither	3,618	96.9	2,090	96.1	1,528	98.1	
Smoking Status (n, %)†							
Current smoker	906	24.3	506	23.3	400	25.7	0.011
Ex-smoker	790	21.2	438	20.1	352	22.6	
Never smoker	2,037	54.5	1,232	56.6	805	51.7	

Nutritional characteristics, and omega-6 and omega-3 dietary intake

Table 2 illustrates the total EI, macronutrients, and omega-6 and omega-3 dietary intakes for the whole sample and by gender. Males were found to have more EI than females by 504.7 kcal (95%CI: 472.2, 537.1) and this difference was significant. The proportions of EI obtained from protein and carbohydrates were significantly higher in males than females. While the % of EI from total fat, SFA, MUFAs, and trans fatty acids (TFA) were significantly higher in females than males, using adjusted regression for important confounders. The proportions of EI obtained from omega-6 and omega-3 were higher among females than males. However, the average ratio of Σω-6/Σω-3 was 5.5:1 ± 2 and there were no significant differences by gender.

**Table 2:** Total energy intake and macronutrients for the whole study sample and by gender EI, energy intake ^†^Regression model-1 adjusted for age, EI, BMI, total house income, ethnicity, and smoking status.

Dietary characteristics	All Participants (n= 3,733)		Females (n=2,176)		Males (n=1,557)		Differences between gender (female is the reference); Coefficient. (95%CI)^†^	P-value
	Mean	±SD	Mean	±SD	Mean	±SD		
Total energy (Kcal)	1823	557.74	1614.32	445.45	2115.1	567.85	-504.7 (-537.1, -472.2)	< 0.001
Protein (g)	73.66	23.45	65.71	19.14	84.76	24.39		
% EI from protein	17.32	3.95	17.29	3.96	17.38	3.94	-1.07 (-1.34, -0.79)	< 0.001
Carbohydrate (g)	219.20	70.81	196.06	57.91	251.54	74.45		
% EI from carbohydrate	47.66	6.93	47.73	6.98	47.55	6.86	-0.81 (-1.31, -0.31)	0.001
Fat (g)	68.31	26.09	61.16	22.91	78.29	26.97		
% EI from fat	35.01	6.42	34.96	6.65	35.10	6.09	1.87 (1.43, 2.32)	< 0.001
% EI from saturated fatty acids	12.88	3.39	12.87	3.52	12.89	3.22	0.95 (0.71, 1.18)	< 0.001
% EI from monounsaturated fatty acids	12.83	2.89	12.72	2.99	12.97	2.75	0.49 (0.29, 0.70)	< 0.001
% EI from trans fatty acids	0.58	0.28	0.56	0.28	0.55	0.26	0.03 (0.01, 0.05)	0.010
Σω-6 (g)	9.76	4.33	8.84	3.94	11.05	4.51		
% EI from Σω-6	5.0	1.55	5.09	1.66	4.97	1.40	0.27 (0.16, 0.38)	< 0.001
Σω-3 (g)	1.94	1.02	1.76	0.94	2.19	1.08		
% EI from Σω-3	1.01	0.45	1.02	0.47	0.99	0.41	0.10 (0.02, 0.09)	0.001
Σω-6/Σω-3 ratio	5.54	2.12	5.55	2.22	5.52	1.99	-0.06 (-0.21, 0.09)	0.466

Association of general and abdominal obesity with omega-6 to omega-3 ratio

There was a significant association between the ratio of Σω-6/Σω-3 fifth quintiles and BMI, WC, WHtR, and RFM (Table 3). The percentage of total EI from total fat was only significant with BMI, while the percentage of EI from omega-3 was negatively associated with WC, WHtR, and RFM. However, the proportion of EI from omega-6 was not associated with any of the obesity indicators.

**Table 3:** Effect of the percentage of EI from total fat, omega-6, omega-3 and quintiles of Σω-6/Σω-3 ratio in general and abdominal obesity ^†^Regression model-2 adjusted to sex, age, EI, total house income, ethnicity and smoking status; *Q is the ratio quintile (mean± SD), and Q1 is the reference category.

	General and abdominal obesity (n = 3,733)							
	BMI (kg/m^2^)		WC (cm)		WHtR		RFM	
	Coef., 95%CI	P-value^†^	Coef., 95%C	P-value^†^	Coef., 95%CI	P-value^†^	Coef., 95%CI	P-value^†^
% EI from total fat	-0.04 (-0.07, -0.01)	0.010	-0.02 (-0.09, 0.05)	0.556	-0.002 (-0.001, 0.002)	0.412	-0.02 (-0.05, 0.01)	0.166
% EI from Σω-6	0.04 (-0.12, 0.11)	0.938	0.17 (-0.11, 0.45)	0.229	0.01(-0.001, 0.002)	0.197	0.07 (-0.04, 0.18)	0.201
% EI from Σω-3	-0.36 (-0.76, 0.04)	0.077	-1.10 (-2.1, -0.12)	0.028	-0.01 (-0.01, -0.01)	0.023	-0.42 (-0.81, -0.10)	0.036
Σω-6/Σω-3								
Q1* (3.1±0.71)	1	0.050	1	0.006	1	0.007	1	0.020
Q2 (4.5±0.24)	0.64 (0.10, 1.17)		2.1 (0.76, 3.42)		0.01 (0.03, 0.02)		0.74 (0.21,1.27)	
Q3 (5.3±0.22)	0.71 (0.25, 1.20)		2.3 (0.95, 3.60)		0.14 (0.01, 0.02)		0.81 (0.28, 1.35)	
Q4 (6.2±0.34)	0.20 (0.01, 0.93)		1.7 (0.33, 2.97)		0.01 (0.01, 0.02)		0.55 (0.03, 1.10)	
Q5 (8.57±2.3)	0.46 (0.08, 1.01)		1.7 (0.39, 3.03)		0.01 (0.01, 0.20)		0.66 (0.13, 1.19)	

## Discussion

The present study assessed the association between PUFAs, particularly omega-6, and omega-3, and the ratio of omega-6/omega-3, with general and abdominal obesity parameters. The findings indicate that the proportion of EI from total fat was only linked with BMI, while the proportion of EI from omega-3 was inversely associated with WC, WHtR, and RFM. In addition, the Σω-6/Σω-3 ratio was associated positively with all selected obesity indicators. The overall ratio Σω-6/Σω-3 was 5.5 ± 2. However, the proportion of EI from omega-6 was not associated with any of the obesity indicators.

Recently, the intake of omega-6 fatty acids has increased, and the intake of omega-3 has decreased, resulting in a large increase in the ratio of omega-6 to omega-3 in Western diets [24]. These changes in the composition of fatty acids are due to agribusiness and modern agriculture and have occurred in parallel with a significant increase in the prevalence of overweight and obesity [13].

Omega-6 and omega-3 are essential fatty acids that must be derived from a person’s diet, as the human body and other mammals cannot make them as a result of a lack of endogenous enzymes for omega-3 desaturation [28]. A mixture of PUFAs (omega-6 and omega-3) should provide an average of 6% of the total EI recommended dietary intake [38]. A balance in the diet between omega-6 and omega-3 fatty acids is very important, as they are metabolically and functionally different, and often have important opposing physiological effects [13,24,28].

The findings of this study showed that an imbalance between dietary intakes of omega-6 and omega-3 was positively associated with general and abdominal obesity. In line with this finding, general obesity and overweight were negatively associated with the Σω-3/Σω-6 ratio after adjustment for important covariates [8]. A linear regression model showed that WC, insulin, and Homeostatic model (assessment of insulin resistance) had positive associations with dietary Σω-6/Σω-3 ratio [39]. Data showed that a high ratio of Σω-6/Σω-3 increased inflammation and chronic inflammatory syndromes, including obesity, fatty liver syndrome, and mortality [40].

The NIH Women’s health study, which presented the importance of omega-6 and omega-3 in weight gain over a mean follow-up period of 10 years, found that increasing the ω6/ω3 ratio increased the risk of becoming overweight or obese at any follow-up time point [41]. The findings of the NIH Women’s health study provide important evidence, as it used an omega-3 index (measuring the concentration of omega-3 in red blood cell membrane phospholipids, assessing medium-term body intake of omega-3 for a period of three months) to find that high omega-3 index was associated with decreasing risk of obesity [41]. Similarly, in a study of another population group, it was found that a low omega-3 index was associated with higher BMI, weight, and WC [19,42].

The findings from this study show that the proportion of EI from omega-3 was inversely associated with WC, WHtR, and RFM and there is a trend for association with BMI. Many studies have found a significant inverse correlation between omega-3 status (either in plasma or omega-3 index) and BMI or obesity [19,20,42]. Another study suggested that a higher omega-3 status can be considered protective against obesity, as it increases basal fat oxidation and so reduces overall fat mass and BMI [43]. On the other hand, no association was found in this study between the proportion of EI from omega-6 and BMI, WC, WHtR, and RFM. This is in line with the findings of the study covering Ghana, where neither omega-6 nor omega-3 was shown to be related to quintiles of BMI, WC, of WHtR [8]. Some studies have emphasized the role of omega-6 in terms of its procardiogenic properties. One found that rodents fed on diets containing 6%-8% of EI from omega-6, similar to the US diet, had increased fat mass [44]. In addition, data from the study found that SFA was only associated with general obesity, while TFA was only linked with WC and no association was found between MUFA and general or abdominal obesity (unpublished results).

Our results emphasize the importance of the ratio of Σω-6/Σω-3 more than the individual PUFA. A lower Σω-6/Σω-3 ratio should be reputed in the management of overweight and obesity with increasing consumption of omega-3 [13,26]. The consequences of high omega-6 remain controversial, as it has intrinsic cardiovascular protective actions, modifying the latest FAO/WHO recommendations on maintaining omega-6 intake if omega-3 intake is high [45]. Furthermore, clinical trials provide conflicting findings on both positive [46] and negative [47] effects of using omega-3 as a weight-reducing agent [13]. Omega-6 and omega-3 are not interconvertible, being metabolically and functionally different. Omega-6 increases adipose tissue and white adipose tissue and reduces brown adipose, as arachidonic acid (AA) prevents the browning of white adipose tissue which considers desirable to avoid weight gain [48]. While dietary omega-3 decreases adipose tissue and white adipose tissue and increases brown adipose tissue, therefore it can lead to weight loss. Thus, a high dietary intake of omega-6 leads to an increase in white adipose tissue and chronic inflammation [24,49]. It is therefore important to balance omega-6 and omega-3 in the diet in order to manage body weight.

The present study has some limitations. The value of omega-6 and omega-3 reported in the NDNS-RP was based on four-day food diaries and there was no information about their concentrations in red blood cell membrane phospholipids. However, the study by Amiano et al. shows that there was a statistically significant relationship between dietary intake and serum levels of omega-3 PUFA, EPA and DHA [50]. A study that measures the dietary intake and obtains actual measurements of plasma or omega-3 and omega-6 in red blood cell membrane phospholipids with information regarding genetic variants to obesity would help to clarify these results and set specific values for the omega-6 to omega-3 ratio. To date, there are no formulated dietary reference values (DRVs) for the intake of PUFAs, especially for omega-6 have not been determined [51]. Only an adequate intake for linoleic acid of 4% EI has been set based on the lowest estimated mean intake of groups of the European population [51].

The present study has various important strengths. First, it is based on up-to-date NDNS-RP data, which are considered very high-quality data. Nationally representative nutritional data for the UK is collected using large sample size and covering seasonal variations in dietary intake. Also, the exclusion of participants with very low or high EI has taken place along with adjustments made for important confounders with the aim of achieving better results. Furthermore, in this study different obesity indicators have been used (general and abdominal obesity), as BMI alone would miss the 35% of men and 14% of women who have normal BMI but central fat distribution. Thus, WHtR has been used as it is considered a simple and effective screening tool for cardiovascular disease risk factors [34,35]. Also, RFM is more accurate than BMI to estimate whole body fat percentage among men and women and has improved obesity misclassification [36].

## Conclusions

Obesity is a preventable disease that can be treated through a healthy diet and lifestyle. Results suggest that the effect of omega-6 and omega-3 PUFA intake reflected in the ratio may be largely driven by a deficiency in the intake of omega-3 fatty acids in increasing general and abdominal obesity among adults. Due to omega-6 and omega-3 fatty acids being physiologically and metabolically distinct and having opposing properties, they should be listed as such in clinical studies and national surveys instead of PUFA. Until more precise dietary recommendations are developed for omega-6 and omega-3 and the appropriate ratio for health is determined, public health practitioners and nutritional counselors should focus on meeting recommended levels of omega-3 and lowering the Σω-6/Σω-3 ratio in order to establish healthier dietary patterns that may positively impact on managing obesity. The effect of omega-6 and omega-3 essential fatty acids on general and abdominal obesity should be further studied using a cohort study.

## References

[1] Kelly T, Yang W, Chen CS, Reynolds K, He J (2008). Global burden of obesity in 2005 and projections to 2030. Int J Obes (Lond).

[2] Baker C (2021). Obesity Statistics Briefing Paper. https://dera.ioe.ac.uk/id/eprint/37120.

[3] Blüher M (2013). Adipose tissue dysfunction contributes to obesity related metabolic diseases. Best Pract Res Clin Endocrinol Metab.

[4] Grundy SM (2016). Metabolic syndrome update. Trends Cardiovasc Med.

[5] Phillips CM, Kesse-Guyot E, McManus R, Hercberg S, Lairon D, Planells R, Roche HM (2012). High dietary saturated fat intake accentuates obesity risk associated with the fat mass and obesity-associated gene in adults. J Nutr.

[6] Moleres A, Ochoa MC, Rendo-Urteaga T, Martínez-González MA, Azcona San Julián MC, Martínez JA, Marti A (2012). Dietary fatty acid distribution modifies obesity risk linked to the rs9939609 polymorphism of the fat mass and obesity-associated gene in a Spanish case-control study of children. Br J Nutr.

[7] Raatz SK, Conrad Z, Johnson LK, Picklo MJ, Jahns L (2017). Relationship of the reported intakes of fat and fatty acids to body weight in US adults. Nutrients.

[8] Suara SB, Siassi F, Saaka M, Foroshani AR, Asadi S, Sotoudeh G (2020). Dietary fat quantity and quality in relation to general and abdominal obesity in women: a cross-sectional study from Ghana. Lipids Health Dis.

[9] Ratnayake WM, Galli C (2009). Fat and fatty acid terminology, methods of analysis and fat digestion and metabolism: a background review paper. Ann Nutr Metab.

[10] Forouhi NG, Sharp SJ, Du H (2009). Dietary fat intake and subsequent weight change in adults: results from the European Prospective Investigation into Cancer and Nutrition cohorts. Am J Clin Nutr.

[11] Soares MJ, Piers LS, Walker KZ, O'Dea K (2003). Is there a role for monounsaturated fat in the dietary management of obesity?. Asia Pac J Public Health.

[12] Molendi-Coste O, Legry V, Leclercq IA (2011). Why and how meet n-3 PUFA dietary recommendations?. Gastroenterol Res Pract.

[13] Simopoulos AP (2016). An increase in the omega-6/omega-3 fatty acid ratio increases the risk for obesity. Nutrients.

[14] Amri EZ, Ailhaud G, Grimaldi PA (1994). Fatty acids as signal transducing molecules: involvement in the differentiation of preadipose to adipose cells. J Lipid Res.

[15] James MJ, Gibson RA, Cleland LG (2000). Dietary polyunsaturated fatty acids and inflammatory mediator production. Am J Clin Nutr.

[16] Schwinkendorf DR, Tsatsos NG, Gosnell BA, Mashek DG (2011). Effects of central administration of distinct fatty acids on hypothalamic neuropeptide expression and energy metabolism. Int J Obes (Lond).

[17] Swanson D, Block R, Mousa SA (2012). Omega-3 fatty acids EPA and DHA: health benefits throughout life. Adv Nutr.

[18] Martínez-Fernández L, Laiglesia LM, Huerta AE, Martínez JA, Moreno-Aliaga MJ (2015). Omega-3 fatty acids and adipose tissue function in obesity and metabolic syndrome. Prostaglandins Other Lipid Mediat.

[19] Micallef M, Munro I, Phang M, Garg M (2009). Plasma N-3 polyunsaturated fatty acids are negatively associated with obesity. Br J Nutr.

[20] Mingay E, Veysey M, Lucock M (2016). Sex-dependent association between omega-3 index and body weight status in older Australians. J Nutrit Intermediary Metabol.

[21] Innes JK, Calder PC (2018). Omega-6 fatty acids and inflammation. Prostaglandins Leukot Essent Fatty Acids.

[22] Dorajoo R, Sun Y, Han Y (2015). A genome-wide association study of n-3 and n-6 plasma fatty acids in a Singaporean Chinese population. Genes Nutr.

[23] Sanders TA (2000). Polyunsaturated fatty acids in the food chain in Europe. Am J Clin Nutr.

[24] Simopoulos AP, DiNicolantonio JJ (2016). The importance of a balanced ω-6 to ω-3 ratio in the prevention and management of obesity. Open Heart.

[25] Blasbalg TL, Hibbeln JR, Ramsden CE, Majchrzak SF, Rawlings RR (2011). Changes in consumption of omega-3 and omega-6 fatty acids in the United States during the 20th century. Am J Clin Nutr.

[26] Simopoulos AP (2011). Evolutionary aspects of diet: the omega-6/omega-3 ratio and the brain. Mol Neurobiol.

[27] Ervin RB, Wright JD, Wang CY, Kennedy-Stephenson J (2004). Dietary intake of fats and fatty acids for the United States population: 1999-2000. Adv Data.

[28] Wijendran V, Hayes KC (2004). Dietary n-6 and n-3 fatty acid balance and cardiovascular health. Annu Rev Nutr.

[29] Wang L (2016). Omega-3 and omega-6 fatty acids: Role in body fat gain and development of obesity. North Am J Med Sci.

[30] (2021). Public Health England, Collection National Diet and Nutrition Survey. User Guide for UK Data. PHE.

[31] Bates BL, A.O. A.O., A.Gay A.Gay, C. Prentice, A. Bates, C. Swan (2021). National Diet and Nutrition Survey: Appendix B Methodology for Year 9 of the NDNS RP. https://s3.eu-west-2.amazonaws.com/fsa-catalogue2/NDNS+Y1-9_Appendix+B_Methodology_FINAL.pdf.

[32] Willett W (1998). Issues in analysis and presentation of dietary data. Nutr epidemiol.

[33] (2021). Obesity: The Prevention, Identification, Assessment and Management of Overweight and Obesity in Adults and Children. https://pubmed.ncbi.nlm.nih.gov/22497033/.

[34] Ashwell M, Gibson S (2016). Waist-to-height ratio as an indicator of 'early health risk': simpler and more predictive than using a 'matrix' based on BMI and waist circumference. BMJ Open.

[35] Ashwell M, Gibson S (2009). Waist to height ratio is a simple and effective obesity screening tool for cardiovascular risk factors: analysis of data from the British National Diet and Nutrition Survey of adults aged 19-64 years. Obes Facts.

[36] Woolcott OO, Bergman RN (2018). Relative fat mass (RFM) as a new estimator of whole-body fat percentage ─ a cross-sectional study in American adult individuals. Sci Rep.

[37] StataCorp. StataCorp. (2013). Statistical Software Release 13. https://www.stata.com/support/faqs/resources/citing-software-documentation-faqs/.

[38] (1991). Dietary reference values for food energy and nutrients for the United Kingdom. Report of the Panel on Dietary Reference Values of the Committee on Medical Aspects of Food Policy. Rep Health Soc Subj (Lond).

[39] Torres-Castillo N, Silva-Gómez JA, Campos-Perez W (2018). High dietary ω-6:ω-3 PUFA ratio is positively associated with excessive adiposity and waist circumference. Obes Facts.

[40] Seiler A, Chen MA, Brown RL, Fagundes CP (2018). Obesity, dietary factors, nutrition, and breast cancer risk. Curr Breast Cancer Rep.

[41] Wang L, Manson JE, Rautiainen S, Gaziano JM, Buring JE, Tsai MY, Sesso HD (2016). A prospective study of erythrocyte polyunsaturated fatty acid, weight gain, and risk of becoming overweight or obese in middle-aged and older women. Eur J Nutr.

[42] Burrows T, Collins CE, Garg ML (2011). Omega-3 index, obesity and insulin resistance in children. Int J Pediatr Obes.

[43] Couet C, Delarue J, Ritz P, Antoine JM, Lamisse F (1997). Effect of dietary fish oil on body fat mass and basal fat oxidation in healthy adults. Int J Obes Relat Metab Disord.

[44] Muhlhausler BS, Ailhaud GP (2013). Omega-6 polyunsaturated fatty acids and the early origins of obesity. Curr Opin Endocrinol Diabetes Obes.

[45] Visioli F, Poli A (2020). Fatty acids and cardiovascular risk. Evidence, lack of evidence, and diligence. Nutrients.

[46] Hill AM, Buckley JD, Murphy KJ, Howe PR (2007). Combining fish-oil supplements with regular aerobic exercise improves body composition and cardiovascular disease risk factors. Am J Clin Nutr.

[47] Chan DC, Watts GF, Nguyen MN, Barrett PH (2006). Factorial study of the effect of n-3 fatty acid supplementation and atorvastatin on the kinetics of HDL apolipoproteins A-I and A-II in men with abdominal obesity. Am J Clin Nutr.

[48] Pisani DF, Ghandour RA, Beranger GE (2014). The ω6-fatty acid, arachidonic acid, regulates the conversion of white to brite adipocyte through a prostaglandin/calcium mediated pathway. Mol Metab.

[49] Pisani DF, Amri E-Z, Ailhaud G (2015). Disequilibrium of polyunsaturated fatty acids status and its dual effect in modulating adipose tissue development and functions. OCL.

[50] Amiano P, Dorronsoro M, de Renobales M, Ruiz de Gordoa JC, Irigoien I (2001). Very-long-chain omega-3 fatty acids as markers for habitual fish intake in a population consuming mainly lean fish: the EPIC cohort of Gipuzkoa. European Prospective Investigation into Cancer and Nutrition. Eur J Clin Nutr.

[51] EFSA Panel on Dietetic Products, Nutrition Nutrition, and Allergies (2010). Scientific opinion on dietary reference values for fats, including saturated fatty acids, polyunsaturated fatty acids, monounsaturated fatty acids, trans fatty acids, and cholesterol. EFSA J.

